# Morphological changes of the tricuspid valve complex in functional tricuspid regurgitation on contrast-enhanced computed tomography

**DOI:** 10.1186/s13019-022-01937-0

**Published:** 2022-08-19

**Authors:** Hiroki Uchiyama, Kazutoshi Tachibana, Koichi Osuda, Nobuyoshi Kawaharada

**Affiliations:** 1 Department of Cardiovascular Surgery, Sapporo Medical University, Minami 1-jo Nishi 16-chome, Chuo-ku, Sapporo, 060-8543 Japan; 2 Department of Cardiovascular Surgery, Hakodate Goryoukaku Hospital, Hakodate, Japan; 3 Division of Radiology, Hakodate Goryoukaku Hospital, Hakodate, Japan

**Keywords:** Functional tricuspid regurgitation, Contrast-enhanced computed tomography, Tricuspid annulus area, Tricuspid annulus circumference, Right ventricular volume, Distance between papillary muscles

## Abstract

**Background:**

Whether it is possible to perform morphological evaluation of functional tricuspid regurgitation (FTR) on contrast-enhanced computed tomography (CT) was examined by evaluating the relationships between the parameters measured on contrast-enhanced CT and TR severity on transthoracic echocardiography.

**Methods:**

Fifty patients underwent contrast-enhanced CT. Tricuspid annulus area (TAA), tricuspid annulus circumference (TAC), right ventricular volume (RVV), and the distances between the tips and bases of the papillary muscles were measured on contrast-enhanced CT in diastole and systole. The 50 cases were divided into 34 in the TR ≤ mild group (no TR: 3 cases, trivial TR: 24 cases, mild TR: 7 cases), and 16 in the TR ≥ moderate group (moderate TR: 8 cases, severe TR: 8 cases) using the TR grade measured by transthoracic echocardiography, and then differences between the groups were examined.

**Results:**

Significant differences were found in TAA, TAC, and RVV (*p* < 0.01) and the distances between the tips of the anterior and posterior papillary muscles (*p* < 0.05) in both diastole and systole. Since the septal papillary muscle could not be identified in 18 cases (36.0%), only the distance between the anterior and posterior papillary muscles was measurable in all cases. On receiver-operating characteristic (ROC) curve analysis, the areas under the ROC curves (AUCs) of TAA, TAC, and RVV were all > 0.7, and the maximum AUC was 0.925 for dRVV.

**Conclusions:**

TAA, TAC, RVV, and the distance between the tips of the anterior and posterior papillary muscles measured on contrast-enhanced CT were shown to be significantly increased in the TR ≥ moderate group. Detailed morphological assessment of FTR is possible by contrast-enhanced CT.

## Background

The tricuspid valve has long been called “the forgotten valve” or “the silent valve”, and the importance of tricuspid valve disease has been undervalued compared with left-sided valve diseases. However, the tricuspid valve is composed of leaflets (anterior leaflet, posterior leaflet, and septal leaflet), an annulus, chordae tendineae, papillary muscles (anterior papillary muscle, posterior papillary muscle, septal papillary muscle), right atrium, and right ventricle. Since it is a complicated structure, it has been found that it profoundly affects right ventricular function, and that residual or worsening tricuspid regurgitation (TR) also leads to worsening quality of life (QOL) and prognosis [1].

Functional tricuspid regurgitation (FTR) occurs due to morphological changes of the tricuspid valve complex that develop secondary to tricuspid annulus dilation and ventricular enlargement as a result of volume or pressure overload of the right ventricle due to left-sided valve diseases [2–5].

FTR severity is diagnosed by transthoracic echocardiography (TTE) and estimated semiquantitatively using the range or area of the regurgitant jet. TTE is performed easily, and a past study evaluated morphological changes of the tricuspid valve complex by TEE [6]. However, TEE has some limitations, such as the need for the evaluator to have experience and the restriction of ultrasound examination by ribs or air.

On the other hand, contrast-enhanced computed tomography (CT) is performed easily and is useful for morphological assessment. Recently, detailed preoperative morphological assessment using contrast-enhanced CT has been increasing, for example, for transcatheter aortic valve implantation (TAVI) [7–9]. However, few papers have considered morphological assessment of FTR using contrast-enhanced CT.

We postulated that, in FTR, morphological changes of tricuspid annulus area, tricuspid annulus circumference, right ventricular volume, and distance between papillary muscles could be identified by contrast-enhanced CT.

In this study, whether it is possible to perform morphological evaluation of FTR on contrast-enhanced CT was examined by evaluating the relationships between the parameters measured by contrast-enhanced CT and TR severity on TTE.

## Methods

### Study population

Between April 2018 and April 2020, 50 patients planned for cardiovascular surgery underwent contrast-enhanced CT and TTE. Patients who had de novo myocardial infarction within less than 28 days, unstable angina, end-stage renal failure, infective endocarditis, active hemorrhagic diseases (gastrointestinal bleeding, trauma, etc.), or postoperative pacemaker implantation were excluded. This study was approved by the research ethics committee of Sapporo Medical University.

The 50 cases were divided into two groups by TR grade measured by TR jet area on TTE, the TR ≤ mild group and the TR ≥ moderate group, and then differences between the groups were examined.

### Contrast-enhanced computed tomography

ECG-gated 320-detector-row multislice computed tomography (Aquilion one, Toshiba Medical Systems, Tokyo, Japan) was used for this study. In order to ensure that the tricuspid annulus and right ventricle would be clearly depicted, very early phase images were taken.

**Fig. 1:**
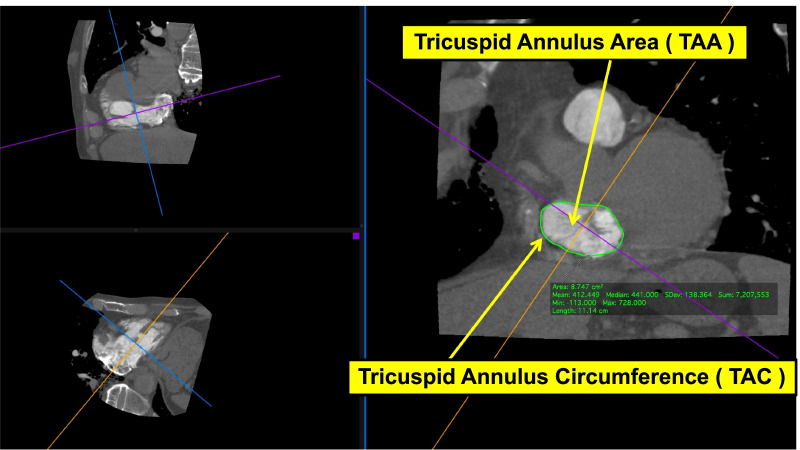
Tricuspid annulus area (TAA) and tricuspid annulus circumference (TAC)

**Fig. 2:**
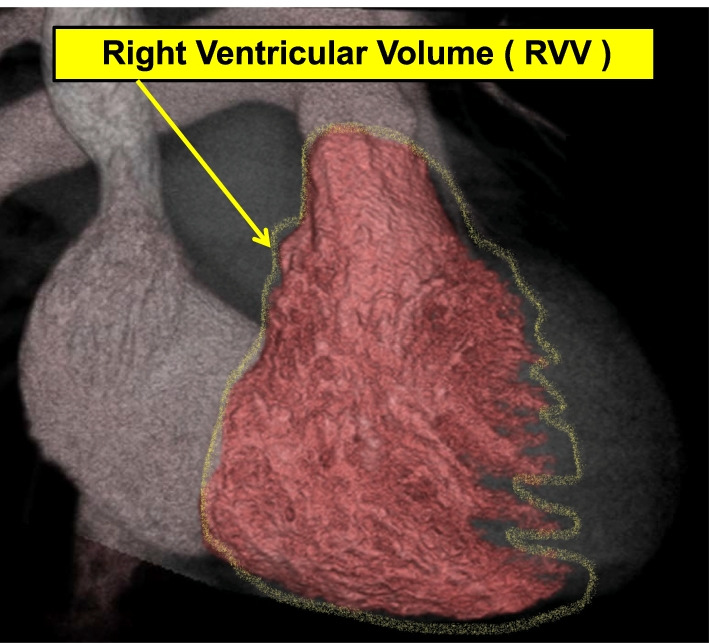
Right ventricular volume (RVV)

**Fig. 3:**
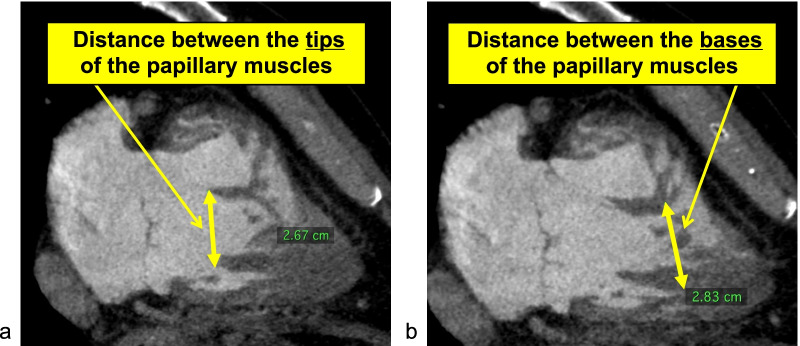
**a** Distance between the tips of the papillary muscles, **b** Distance between the bases of the papillary muscles

The reconstructed volume data images were transferred to OsiriX (Pixmeo, Geneva, Switzerland) and Ziostation2 (Ziosoft, Tokyo, Japan). Tricuspid annulus area (TAA), tricuspid annulus circumference (TAC), right ventricular volume (RVV), and the distances between the tips (t) and bases (b) of the papillary muscles (anterior, posterior, and septal) were measured on contrast-enhanced CT. Each was measured at diastole (d) and at systole (s). Figure 1 shows the measurement chart using OsiriX for the tricuspid annulus area, the perimeter of the tricuspid annulus, and tricuspid annulus circumference. Figure 2 shows the measurement chart using Ziostation2 for right ventricular volume, and Fig. 3 shows the measurement chart using OsiriX for the distances between the tips and bases of the papillary muscles.

One researcher who was aware of the group allocation of the patients performed all of the measurements.

### Transthoracic echocardiography

The Philips iE33 (Koninklijke Philips N.V., Amsterdam, The Netherlands) was used in this study. In addition to the general TTE measurements (ejection fraction (EF), aortic diameter (AOD), left atrial diameter (LAD), left ventricular end diastolic diameter (LVDd), left ventricular end systolic diameter (LVDs), interventricular septal thickness at end diastole (IVSTD), posterior wall thickness at end diastole (PWTD), end diastolic volume (EDV), end systolic volume (ESV)), tricuspid regurgitation grade (jet area < 5cm^2^: TR ≤ mild, and jet area ≥ 5cm^2^: TR ≥ moderate), tricuspid annulus diameter (TA, end-diastole, 4-chamber view), vena contracta (VC), jet area (JA), tricuspid annulus plane systolic excursion (TAPSE), functional area change (FAC), predicted right ventricular pressure (RVP), and inferior vena cava diameter (IVC) were also measured. The prevalence of atrial fibrillation was evaluated.

### Statistical analysis

Continuous variables are expressed as means ± standard deviation (SD), and categorical variables are expressed as frequencies (percent). For continuous variables, normality was assessed with the Shapiro–Wilk test, and homogeneity of variance was determined by the Levene test. Student’s *t-*test (for homogeneity of variance), Welch’s test (for non-homogeneity of variance), or the Mann–Whitney U test (for non-normal distribution) was used to compare continuous variables. Categorical variables were compared by the χ^2^ test. *p* < 0.05 was considered significant.

Receiver-operating characteristic (ROC) curve analysis was used to evaluate the relationships between parameters measured on contrast-enhanced CT and TR grade measured on TTE and to determine the best critical value and its sensitivity and specificity.

All statistical analyses were performed using IBM SPSS Statistics version 26.0 (IBM, Armonk, NY, USA).

## Results

Table 1 shows the patients’ characteristics and the parameters of TTE for each TR grade. Of the 50 cases, 34 were in the TR ≤ mild group (no TR: 3 cases, trivial TR: 24 cases, mild TR: 7 cases), and 16 were in the TR ≥ moderate group (moderate TR: 8 cases, severe TR: 8 cases). There were no significant differences in age, sex, or body surface area between the two groups. Of the TTE parameters, significant differences were observed in LAD and IVSTD.

**Table 1:** Patients’ characteristics and parameters measured by transthoracic echocardiography

Variable^a^	All cases (n = 50)	TR ≤ mild (n = 34)	TR ≥ moderate (n = 16)	*p*-value
*Patients’ characteristics*				
Age (y)	73.6 ± 9.2	72.3 ± 10.2	76.5 ± 5.7	0.066
Male sex, n (%)	31 (62.0)	22 (64.7)	9 (56.3)	0.566
BSA (cm^2^)	1.59 ± 0.16	1.60 ± 0.15	1.57 ± 0.19	0.569
*Transthoracic echocardiography*				
EF (%)	64.3 ± 10.1	63.5 ± 10.8	65.9 ± 8.6	0.574
AOD (mm)	33.7 ± 5.2	33.9 ± 5.4	33.3 ± 5.1	0.702
LAD (mm)	43.3 ± 9.2	40.7 ± 8.0	48.9 ± 9.4	0.003
LVDd (mm)	48.3 ± 7.5	48.2 ± 7.7	48.4 ± 7.2	0.851
LVDs (mm)	31.8 ± 9.2	32.5 ± 9.1	30.4 ± 9.6	0.956
IVSTD (mm)	10.6 ± 1.9	11.1 ± 2.0	9.6 ± 1.4	0.013
PWTD (mm)	10.2 ± 1.7	10.5 ± 1.7	9.5 ± 1.5	0.072
EDV (mL)	99.9 ± 36.6	106.5 ± 37.1	86.4 ± 32.3	0.071
ESV (mL)	37.7 ± 23.6	41.5 ± 26.5	29.7 ± 13.3	0.090
Af rhythm, n (%)	11 (22.0)	5 (14.7)	6 (37.5)	0.070
Asynergy, n (%)	4 (8.0)	1 (2.9)	3 (18.8)	0.055
TA (mm)	31.8 ± 6.1	29.1 ± 4.0	36.0 ± 6.4	< 0.001
VC (mm)	5.8 ± 4.2	3.4 ± 1.0	8.1 ± 4.8	< 0.001
JA (cm^2^)	6.0 ± 6.3	1.7 ± 0.9	10.0 ± 6.6	< 0.001
TAPSE	19.9 ± 3.7	20.5 ± 3.6	19.0 ± 3.8	0.188
FAC (%)	38.6 ± 7.2	39.5 ± 4.8	37.0 ± 10.7	0.472
RVP (mmHg)	30.7 ± 10.9	25.9 ± 6.7	41.6 ± 10.7	< 0.001
RAP (mmHg)	6.5 ± 3.4	5.3 ± 1.8	9.2 ± 4.7	0.001
IVC (mm)	16.4 ± 4.6	15.0 ± 3.2	19.5 ± 5.7	0.008

*EF* ejection fraction, *AOD* aortic diameter, *LAD* left atrial diameter, *LVDd* left ventricular end diastolic diameter, *LVDs* left ventricular end systolic diameter, *IVSTD* interventricular septal thickness at end diastole, *PWTD* posterior wall thickness at end diastole, *EDV* end diastolic volume, *ESV* end systolic volume, *TA* tricuspid annulus diameter (4 chamber view, diastolic), *VC* vena contracta, *JA* jet area, *TAPSE* tricuspid annulus plane systolic excursion, *FAC* functional area change, *RVP* right ventricular pressure, *RAP* right atrial pressure, *IVC* inferior vena cava diameter

^a^Data are presented as means ± SD or frequencies (percent)

**Fig. 4:**
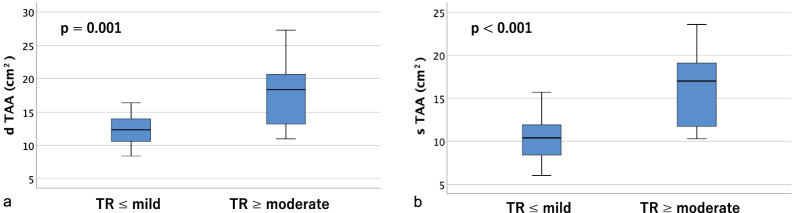
Box-and-whisker plots of diastolic (**a**) and systolic (**b**) tricuspid annulus area (TAA)

**Fig. 5:**
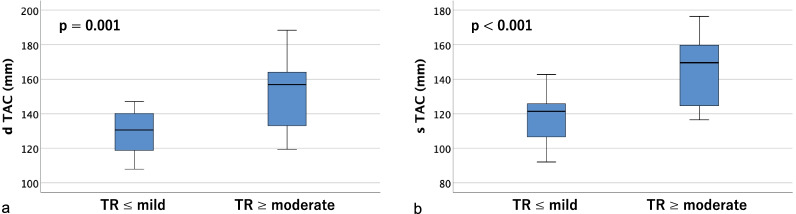
Box-and-whisker plots of diastolic (**a**) and systolic (**b**) tricuspid annulus circumference (TAC)

**Fig. 6:**
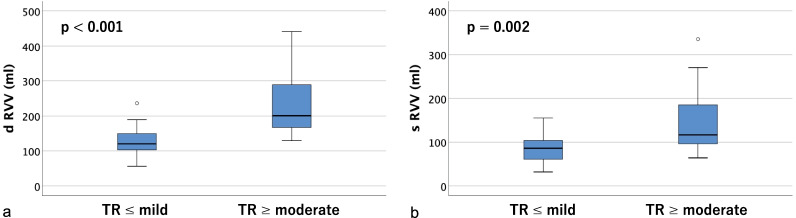
Box-and-whisker plots of diastolic (**a**) and systolic (**b**) right ventricular volumes (RVV)

**Fig. 7:**
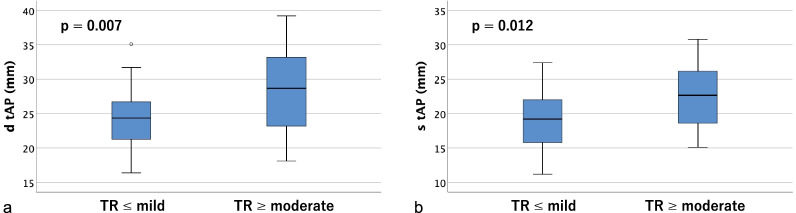
Box-and-whisker plots of diastolic (**a**) and systolic (**b**) distances between the tips of the anterior and posterior papillary muscles (tAP)

**Fig. 8:**
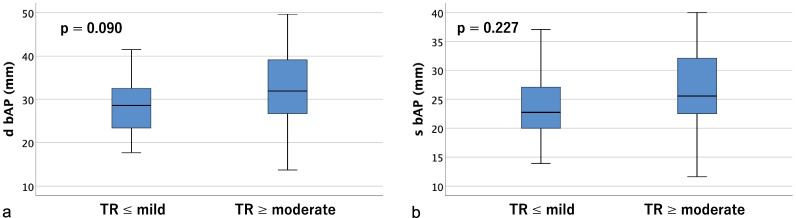
Box-and-whisker plots of diastolic (**a**) and systolic (**b**) distances between the bases of the anterior and posterior papillary muscles (bAP)

Table 2 shows tricuspid annulus area (TAA), tricuspid annulus circumference (TAC), right ventricular volume (RVV), and the distances between the papillary muscles (anterior, posterior, and septal) measured on contrast-enhanced CT. Significant differences were found in tricuspid annulus area (TAA), tricuspid annulus circumference (TAC), and right ventricular volume (RVV) in both diastole and systole (*p* < 0.01). As for the distances between papillary muscles, the anterior and posterior papillary muscles could be identified on contrast-enhanced CT in all cases, but the septal papillary muscle could not be identified in 18 cases (36.0%); therefore, only the distances between the anterior and posterior papillary muscles were measurable in all cases. There were significant differences between groups in the diastolic and systolic distances between the tips of the anterior and posterior papillary muscles (dtAP and stAP), diastolic distance between the tips of the posterior and septal papillary muscles (dtPS), and the diastolic and systolic distances between the bases of the posterior and septal papillary muscles (dbPS and sbPS) (*p* < 0.05). Box-and-whisker plots and p-values for the two groups are shown (tricuspid annulus area in Fig. 4, tricuspid annulus circumference in Fig. 5, right ventricular volume in Fig. 6, distances between the tips of the anterior and posterior papillary muscles in Fig. 7, and distances between the bases of the anterior and posterior papillary muscles in Fig. 8).

**Table 2:** Parameters measured on contrast-enhanced CT

Variable^a^	All cases (n = 50)	TR ≤ mild (n = 34)	TR ≥ moderate (n = 16)	*p*-value
*Contrast-enhanced CT*				
d TAA (cm^2^)	14.1 ± 4.2	12.3 ± 2.3	17.7 ± 5.1	0.001
s TAA (cm^2^)	12.3 ± 4.2	10.4 ± 2.4	16.2 ± 4.5	< 0.001
d TAC (mm)	136.5 ± 18.3	129.6 ± 11.5	151.3 ± 21.3	0.001
s TAC (mm)	126.9 ± 20.2	118.3 ± 13.2	145.2 ± 20.6	< 0.001
d RVV (mL)	161.5 ± 75.4	127.4 ± 35.4	233.8 ± 87.3	< 0.001
s RVV (mL)	106.0 ± 56.8	86.8 ± 30.1	146.7 ± 77.1	0.002
d t AP (mm)	25.8 ± 5.3	24.5 ± 4.3	28.7 ± 6.1	0.007
s t AP (mm)	19.9 ± 4.8	18.8 ± 4.5	22.4 ± 4.6	0.012
d b AP (mm)	29.6 ± 7.2	28.4 ± 5.9	32.1 ± 9.2	0.090
s b AP (mm)	24.3 ± 6.2	23.6 ± 5.5	25.9 ± 7.4	0.227
d t PS (mm)	20.9 ± 7.1	17.8 ± 3.3	25.4 ± 8.7	0.007
s t PS (mm)	16.3 ± 6.7	14.1 ± 3.0	19.4 ± 9.2	0.042
d b PS (mm)	26.4 ± 10.0	22.5 ± 6.2	32.1 ± 11.9	0.008
s b PS (mm)	21.4 ± 7.3	18.7 ± 4.6	25.2 ± 8.9	0.011
d t SA (mm)	32.3 ± 5.1	31.3 ± 4.1	33.8 ± 6.1	0.195
s t SA (mm)	26.1 ± 4.9	25.4 ± 3.7	27.0 ± 6.2	0.430
d b SA (mm)	35.7 ± 8.0	34.2 ± 6.6	37.9 ± 9.6	0.327
s b SA (mm)	28.2 ± 6.1	28.1 ± 5.1	28.2 ± 7.6	0.878

*d TAA* diastolic tricuspid annulus area, *s TAA* systolic tricuspid annulus area, *d TAC* diastolic tricuspid annulus circumference, *s TAC* systolic tricuspid annulus circumference, *d RVV* diastolic right ventricular volume, *s RVV* systolic right ventricular volume, *d t AP* diastolic distance between the tips of the anterior and posterior papillary muscles, *s t AP* systolic distance between the tips of the anterior and posterior papillary muscles, *d b AP* diastolic distance between the bases of the anterior and posterior papillary muscles, *s b AP* systolic distance between the bases of the anterior and posterior papillary muscles, *d t PS* diastolic distance between the tips of the posterior and septal papillary muscles, *s t PS* systolic distance between the tips of the posterior and septal papillary muscles, *d b PS* diastolic distance between the bases of the posterior and septal papillary muscles, *s b PS* systolic distance between the bases of the posterior and septal papillary muscles, *d t SA* diastolic distance between the tips of the septal and anterior papillary muscles, *s t SA* systolic distance between the tips of the septal and anterior papillary muscles, *d b SA* diastolic distance between the bases of the septal and anterior papillary muscles, *s b SA* systolic distance between the bases of the septal and anterior papillary muscles

^a^Data are presented as means ± SD or frequencies (percent)

**Fig. 9:**
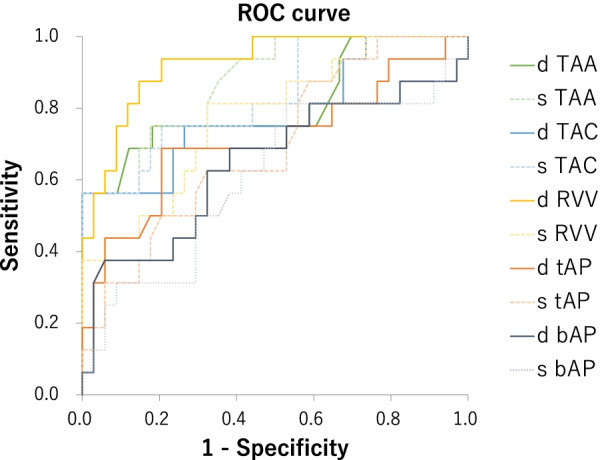
ROC curves of parameters measured on contrast-enhanced CT

The ROC curves of parameters measured on contrast-enhanced CT are shown in Fig. 9. Table 3 shows the area under the ROC curve (AUC), critical value, sensitivity, and specificity of each parameter. AUCs of TAA, TAC, and RVV were all > 0.7, and the maximum AUC was 0.925 for dRVV.

**Table 3:** ROC curve analysis of parameters measured on contrast-enhanced CT

Variable	AUC	Critical value	Sensitivity	Specificity	*p*-value
d TAA (cm^2^)	0.812	14.8	0.750	0.818	< 0.001
s TAA (cm^2^)	0.874	12.5	0.750	0.824	< 0.001
d TAC (mm)	0.787	138.0	0.750	0.735	< 0.001
s TAC (mm)	0.835	127.4	0.750	0.794	< 0.001
d RVV (mL)	0.925	163.2	0.875	0.853	< 0.001
s RVV (mL)	0.778	95.9	0.813	0.677	< 0.001
d t AP (mm)	0.711	27.6	0.688	0.794	0.018
s t AP (mm)	0.694	20.6	0.625	0.677	0.015
d b AP (mm)	0.648	30.8	0.688	0.618	0.114
s b AP (mm)	0.609	24.5	0.625	0.588	0.239

*d TAA* diastolic tricuspid annulus area, *s TAA* systolic tricuspid annulus area, *d TAC* diastolic tricuspid annulus circumference, *s TAC* systolic tricuspid annulus circumference, *d RVV* diastolic right ventricular volume

s RVV: systolic right ventricular volume, *d t AP* diastolic distance between the tips of the anterior and posterior papillary muscles, *s t AP* systolic distance between the tips of the anterior and posterior papillary muscles, *d b AP* diastolic distance between the bases of the anterior and posterior papillary muscles, *s b AP* systolic distance between the bases of the anterior and posterior papillary muscles

## Discussion

The tricuspid annulus area (TAA), tricuspid annulus circumference (TAC), and right ventricular volume (RVV) measured on contrast-enhanced CT were significantly increased in the TR ≥ moderate group, showing that tricuspid annulus dilation and ventricular enlargement are causes of FTR progression. This corroborates the relationships between contrast-enhanced CT parameters and TR grade measured by echocardiography.

According to the 2017 ESC/EACTS guidelines, tricuspid valve surgery for secondary TR is class I for severe TR undergoing left-sided valve surgery, and class IIa for mild or moderate TR with a dilated annulus (≥ 40 mm or > 21 mm/m^2^ on 2D echocardiography) undergoing left-sided valve surgery [10]; in the present study, moderate and severe TR significantly increased tricuspid annulus area (TAA), tricuspid annulus circumference (TAC), and right ventricular volume (RVV) on contrast-enhanced CT. Furthermore, the AUC > 0.7 on ROC analysis also suggests that TAA, TAC, and RVV may be useful for identifying severe TR cases. Therefore, the parameters of contrast-enhanced CT may also be considered in the indications for FTR surgery in the future. On ROC analysis, dRVV showed the maximum AUC. Since the right ventricular volume generally depends on whole body volume, volume management is considered important for TR management.

Regarding the distance between papillary muscles, a significant difference was observed in the distance between the tips of the anterior and posterior papillary muscles, and it is considered that it tends to increase as FTR becomes more severe. In about 1/3 (36.0%) of cases, the septal papillary muscle could not be identified on contrast-enhanced CT. According to autopsy reports, cases with many small septal papillary muscles or tendinous cords that appear directly from the septal walls of the right ventricle have been described, and there are many variations of septal papillary muscles [11]. The findings of the present study were similar, and there were many cases in which septal muscles could not be identified because of the many variations. Anterior and posterior papillary muscles were identified in all cases; only the distances between the anterior and posterior papillary muscles could be measured effectively. In some cases, the posterior papillary muscle was also small, and only the anterior papillary muscle was clearly visible, so it is thought that the anterior papillary muscle contributes most to the tricuspid valve.

Kabasawa and colleagues also evaluated FTR on contrast-enhanced CT [12] in 35 patients who underwent contrast-enhanced CT, and end-diastolic and end-systolic tricuspid valve annular diameters (TVADs), tethering angles, and tethering height were significantly correlated with preoperative TR severity. The result for the tricuspid annulus was similar to that of the present study, and the present study did not evaluate tethering angles and tethering height, but evaluated right ventricular volume and the distances between the papillary muscles.

In comparison with ultrasonography, contrast-enhanced CT is expensive and slightly more invasive because of the use of contrast media and radiation exposure. However, it is convenient, because it can provide morphological evaluations, especially quantitative measurements, easily and in detail. In the present study, the detailed morphological assessment of FTR was possible using contrast-enhanced CT.

In recent years, minimally invasive transcatheter approaches for TR have been introduced, such as the Mitraclip for TR and the FORMA tricuspid repair system as leaflet devices and the Trialign percutaneous tricuspid valve annuloplasty system, the Cardioband tricuspid annuloplasty system, and the TriCinch tricuspid valve repair as annuloplasty devices. Other examples are Transcatheter Tricuspid Valve Replacement (TTVR) and Caval Valve Implantation (CAVI) [13]. In preoperative planning for tricuspid valve treatment in the future, it is likely that evaluation of the tricuspid valve complex on contrast-enhanced CT will be useful. There are reports that CT provides very useful information on annular structure and dimensions, the quality and amount of annular tissue, and its relationship with the right coronary artery [14]

There are several limitations to this study. The tricuspid annulus actually has three-dimensional geometry [15, 16]. However, in the present study, because the tricuspid annulus was measured on a contrast-enhanced CT section, the true annulus structure may not have been evaluated.

Because one researcher who was aware of the group allocations of the patients measured all the data, this could have induced information bias.

The present study did not evaluate the same cases over time, but if they were to be classified according to the surgical intervention and evaluated over time, it may be possible to further deepen our understanding of the morphological changes in FTR and the surgical indications.

## Conclusions

Tricuspid annulus area, tricuspid annulus circumference, right ventricular volume, and the distances between the tips of the anterior and posterior papillary muscles measured on contrast-enhanced CT were shown to be significantly increased in the TR ≥ moderate group.

Detailed morphological assessment of functional tricuspid regurgitation is possible on contrast-enhanced CT.

## Data Availability

The datasets used and/or analyzed during the current study are available from the corresponding author on reasonable request.

## Abbreviations

TR: Tricuspid regurgitation
FTR: Functional tricuspid regurgitation
TTE: Transthoracic echocardiography
CT: Computed tomography
TAA: Tricuspid annulus area
TAC: Tricuspid annulus circumference
RVV: Right ventricular volume
AP: Distance between the anterior and posterior papillary muscles
PS: Distance between the posterior and septal papillary muscles
SA: Distance between the septal and anterior papillary muscles
EF: Ejection fraction
AOD: Aortic diameter
LAD: Left atrial diameter
LVDd: Left ventricular end diastolic diameter
LVDs: Left ventricular end systolic diameter
IVSTD: Interventricular septal thickness at end diastole
PWTD: Posterior wall thickness at end diastole
EDV: End diastolic volume
ESV: End systolic volume
TA: Tricuspid annulus diameter
VC: Vena contracta
JA: Jet area
TAPSE: Tricuspid annulus plane systolic excursion
FAC: Functional area change
RVP: Right ventricular pressure
RAP: Right atrial pressure
IVC: Inferior vena cava diameter
